# Design of multi‐epitope chimeric vaccine against Monkeypox virus and SARS‐CoV‐2: A vaccinomics perspective

**DOI:** 10.1111/jcmm.18452

**Published:** 2024-05-27

**Authors:** Haitham Al‐Madhagi, Adeela Kanawati, Zaher Tahan

**Affiliations:** ^1^ Biochemical Technology Program, Faculty of Applied Sciences Dhamar University Dhamar Yemen; ^2^ Division of Biochemistry, Chemistry Department University of Aleppo Aleppo Syria; ^3^ Division of Microbiology, Biology Department University of Aleppo Aleppo Syria

**Keywords:** COVID‐19, immunoinformatics, monkeypox, SARS‐CoV‐2, vaccine

## Abstract

The current era we experience is full with pandemic infectious agents that no longer threatens the major local source but the whole globe. Almost the most emerging infectious agents are severe acute respiratory syndrome coronavirus‐2 (SARS CoV‐2), followed by monkeypox virus (MPXV). Since no approved antiviral drugs nor licensed active vaccines are yet available, we aimed to utilize immunoinformatics approach to design chimeric vaccine against the two mentioned viruses. This is the first study to deal with design divalent vaccine against SARS‐CoV‐2 and MPXV. ORF8, E and M proteins from Omicron SARS‐CoV‐2 and gp182 from MPXV were used as the protein precursor from which multi‐epitopes (inducing B‐cell, helper T cells, cytotoxic T cells and interferon‐ɣ) chimeric vaccine was contrived. The structure of the vaccine construct was predicted, validated, and docked to toll‐like receptor‐2 (TLR‐2). Moreover, its sequence was also used to examine the immune simulation profile and was then inserted into the pET‐28a plasmid for in silico cloning. The vaccine construct was probable antigen (0.543) and safe (non‐allergen) with strong binding energy to TLR‐2 (−1169.8 kcal/mol) and found to have significant immune simulation profile. In conclusion, the designed chimeric vaccine was potent and safe against SARS‐CoV‐2 and MPXV, which deserves further consideration.

## 1 INTRODUCTION

It was estimated that approximately 95,226 cases worldwide have been infected with monkeypox virus (MPXV) resulting in about 185 deaths.^1^ Before 2022, it had not been deemed an outbreak since it was restricted in few African countries. It invaded many regions throughout the globe from the imports from the endemic sources.^2^ MXPV is an orthopoxvirus emerging zoonotic disease inflecting mankind that belong to Poxviridea family that contains DNA as a genome. Smallpox virus is the closest relative and it exhibits smallpox‐like symptoms.^3^ The natural reservoir of MPXV is not yet identified but sooty mangabey and squirrel are the most suspected.^4^ The transmission from animals to humans are via contacting infected animal or its fluids and organs/products.^5^ MPXV person‐to‐person transmission can be conveyed by respiratory droplets, genital secretions as well as the characteristic skin lesions.^6^ Furthermore, accumulating evidences assured that pregnant mothers can transmit the virus to their foetuses.^7^

MPXV is an enveloped, double‐stranded DNA virus that involves two genetic clades, all of which originated from Africa: Central African (Congo Basin) and West African clades.^8^ In contrast to the West African clade, central African clade is more aggressive since its fatality rate is nearly 11%, compared to 1% for West African clade.^2^ Currently, there are no approved treatments specifically designed to eradicate MPXV. The primary approach is supportive care, particularly for hospitalized patients. With respect to vaccine development, two vaccines are available: JYNNEOS™ (live, replication incompetent vaccinia virus) and ACAM2000® (live, replication competent vaccinia virus).^9^ Such therapy albeit proved potent to some extent, they are repurposed toward and not specific for the emerging MPXV.

On the other hand, Coronavirus disease 2019 (COVID‐19) has infested almost the whole globe, killed more than 6.6 million people worldwide up to date.^10^ Among those, >351 thousand deaths were reported in the Eastern Mediterranean. Paradoxically, low‐income countries like Yemen and Syria were among the least affected by COVID‐19.^11^ Severe acute respiratory syndrome coronavirus‐2 (SARS CoV‐2) is the causative agent of the pandemic. SARS CoV‐2 belongs to positive‐sense, enveloped viruses thereby speeding replication, transcription, translation as well as assembly of new virions and, at the same time, facilitating its fusion process to host cell membranes.^12^ As took place with its counterparts, SARS (severe acute respiratory syndrome) and MERS (Middle‐East respiratory syndrome), SARS‐CoV‐2 may goes through a state of gradual weakness owing to the conserved antigenicity and virulence.^13^ To date, no active licensed vaccine against SARS‐CoV‐2 is available despite the accelerating race between biotech industries and medical institutions. This is due to the highly‐demanding nature of vaccine approval that necessitates long years of experimentation and costly clinical trials.^14^ Recently, the redirection into the exploitation of immunoinformatics as a preceding step of vaccine contriving and testing is a priority as it facilitates the design planning and reduce enormous time and, at the same time, economizes some developmental stages of vaccine manufacturing.^15^

COVID‐19 vaccines have been proven to be highly effective in preventing COVID‐19 infection and reducing the severity of illness, hospitalization, and death. The Pfizer‐BioNTech vaccine has shown an efficacy rate of 95%, while the Moderna vaccine has shown an efficacy rate of 94.1%. Even the AstraZeneca and Johnson & Johnson vaccines, with efficacy rates of 70.4% and 66%, respectively, have been shown to significantly reduce the risk of symptomatic COVID‐19 infection.^16^, ^17^ Furthermore, studies have shown that COVID‐19 vaccines are highly effective in preventing hospitalizations and severe disease. A study conducted in the UK found that the Pfizer‐BioNTech vaccine was 93% effective in preventing hospitalization due to COVID‐19, while the AstraZeneca vaccine was 76% effective. Another report found that the Pfizer‐BioNTech vaccine was 97% effective in preventing severe disease, hospitalization, and death.^18^, ^19^ Importantly, COVID‐19 vaccines have been extensively studied and found to be generally safe. The most common side effects are mild and temporary, such as pain at the injection site, fatigue, headache, and fever. Serious adverse events are rare, and the benefits of vaccination far outweigh the risks.^20^, ^21^, ^22^ Immunoinformatics and reverse vaccinology approaches have been utilized to design a multi‐epitope vaccines against a wide array of highly virulent pathogens which is deemed a preliminary step in the modern biotechnological arena to eradicate such pathogens.^23^, ^24^

As COVID‐19 and MPXV viruses are the most emerging in the recent years, and no radical antiviral nor efficacious vaccines are available, we sought in this study is to design a chimeric vaccine (against SARS‐CoV‐2 and MPXV) using an immunoinformatics approach. This is the first study to deal with design divalent vaccine against SARS‐CoV‐2 and MPXV.

## 2 METHODS

**FIGURE 1:**
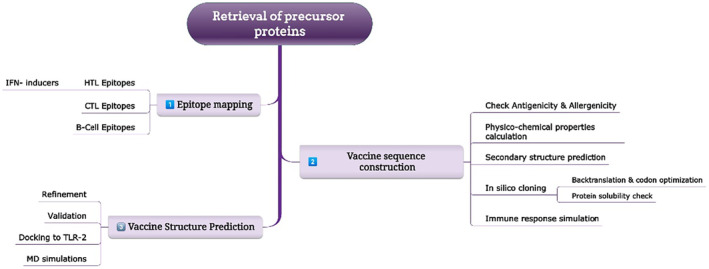
Flowchart representing the general workflow of the present vaccinomics approach adopted in this study.

The strategy deployed in the present study to accomplish the comprehensive design, analysis and testing of the chimeric vaccine is provided in Figure 1.

### 2.1 Sequence retrieval

The genome sequences of SARS‐CoV‐2 (Accession ID #ON249995) and MPXV (Accession ID #NC_063383) were retrieved from the Viralzone web portal. From there, gp182 was used as the antigen of MPXV, while ORF8, envelope (E) proteins, and membrane (M) proteins were used as the antigen for SARS‐CoV‐2.

### 2.2 Epitopes mapping

The selected proteins from the two viruses were examined for the identification of potential immunogenic epitopes. B‐cell epitopes as well as helper T lymphocyte (HTL) epitopes were predicted by (http://tools.iedb.org/bcell/), and (http://tools.iedb.org/mhcii/) (accessed on 28 December 2022).^25^ We selected full HLA reference allele set, i.e. HLA‐DRB1*01:01, HLA‐DRB1*03:01, HLA‐DRB1*04:01, HLA‐DRB1*04:05, HLA‐DRB1*07:01, HLA‐DRB1*08:02, HLA‐DRB1*09:01, HLA‐DRB1*11:01, HLA‐DRB1*12:01, HLA‐DRB1*13:02, HLA‐DRB1*15:01, HLA‐DRB3*01:01, HLA‐DRB3*02:02, HLA‐DRB4*01:01, HLA‐DRB5*01:01, HLA‐DQA1*05:01/DQB1*02:01, HLA‐DQA1*05:01/DQB1*03:01, HLA‐DQA1*03:01/DQB1*03:02, HLA‐DQA1*04:01/DQB1*04:02, HLA‐DQA1*01:01/DQB1*05:01, HLA‐DQA1*01:02/DQB1*06:02, HLA‐DPA1*02:01/DPB1*01:01, HLA‐DPA1*01:03/DPB1*02:01, HLA‐DPA1*01:03/DPB1*04:01, HLA‐DPA1*03:01/DPB1*04:02, HLA‐DPA1*02:01/DPB1*05:01, HLA‐DPA1*02:01/DPB1*14:01. The best ranking HTL epitopes were checked for their capability to induce interferon‐ɣ (IFN‐ ɣ) secretion via IFNepitopes tool (http://crdd.osdd.net/raghava/ifnepitope/).^26^ With respect to cytotoxic T lymphocyte (CTL), its epitopes were predicted using NetCTL 1.2 server (https://services.healthtech.dtu.dk/service.php?NetCTL‐1.2).^27^ All of the predicted epitopes, the corresponding antigenicity, toxicity and allergenicity were checked utilizing VaxiJen 2.0 (http://www.ddg‐pharmfac.net/vaxijen/VaxiJen/VaxiJen.html),^28^, ^29^ ToxinPred (http://crdd.osdd.net/raghava/toxinpred/)^30^ and AllergenFP (https://ddg‐pharmfac.net/AllergenFP/).^31^ Only those epitopes which displayed were found non‐allergen nor toxic were considered for the remaining vaccinomics examination.

### 2.3 Conformational B cell epitopes prediction

The conformational B cell epitopes were also predicted for all antigens using SEMA v2.0 server (https://sema.airi.net/).^32^ The input method was according to PDB ID (7VGR for M protein, 7F5F for ORF8, and 8SUZ for E protein). The visualization of the output involves a colour‐coded protein sequence representation based on predicted values: regions with a low epitope score of zero are depicted in brown, while those exceeding the threshold (0.51) are shown in cyan, indicating them as epitopes. Additionally, amino acids predicted to undergo N‐glycosylation are represented as spheres.

### 2.4 Vaccine construct design and structures prediction

The top‐ranked epitopes were linked together via different linkers such as GPGPG, KK and AAY since these linkers improves the stability of structure, by avoiding self‐folding, enhance the epitope presentation, and boost the immunogenicity of the constructed vaccine.^33^ Moreover, the introduction of an adjuvant in the N‐terminal of the vaccine construct has been demonstrated to better boost the vaccine immunogenicity. Moreover, to facilitate the purification of the vaccine construct after overexpression, His tag (6 His residues) was added to the construct C‐terminal as described earlier.^34^ Afterwards, the vaccine physicochemical properties and secondary structure were predicted via ProtParam (https://web.expasy.org/protparam/)^35^ and PSIPRED (http://bioinf.cs.ucl.ac.uk/psipred/) webservers^36^ while the tertiary structure was modelled using RoseTTafold^37^ and refined by GalaxyRefine server (https://galaxy.seoklab.org/refine).^38^ The refined 3D structure version of the vaccine construct was validated employing PROCHECK and ProsA tools from SAVES server (https://saves.mbi.ucla.edu/).^39^, ^40^

### 2.5 Molecular docking

In order to mimic the binding of a vaccine to the toll‐like receptors (TLR) present on the surface of innate immune cells, protein–protein docking was performed via ClusPro server (https://cluspro.bu.edu/queue.php).^41^ ClusPro is a web server that performs rigid body docking of two proteins by sampling billions of conformations. Low energy docked structures are clustered, and centers of the largest clusters are used as likely models of the complex. TLR‐2 crystal structure was downloaded from PDB (PDB ID#6NIG). The protein–protein interactions were explored by RING 3.0 tool (https://ring.biocomputingup.it/).^42^

### 2.6 Molecular dynamics (MD) simulation

MD simulations of the best docked complex (vaccine‐TLR‐2) were perfromed to infer the stability and flexibility. MD simulation was conducted via the recently developed web portal Visual Dynamics (http://visualdynamics.fiocruz.br/) via its Apo portal.^43^ The operating parameters were set as follows: water model (SPC), box type (cubic), box distance 0.35 mm. The forcefield employed was AMBER 03 for 100 ns. Root mean square deviation (RMSD), root mean square fluctuation (RMSF), and radius of gyration (Rg) were plotted for the MD trajectories of the vaccine‐TLR‐2 complex. Properly folded vaccine protein is fundamental for the effective immune response.^44^ Afterward, we computed free binding energy (MM/GBSA) for vaccine‐TLR‐2 complex.

### 2.7 In silico cloning

The virtual cloning was done using SnapGene software (GSL Biotech; available at snapgene.com). The amino acid sequence of vaccine construct was transformed back to DNA utilizing backtranseq tool of EBML‐EBI web services.^45^ Afterwards, the DNA codon sequence was optimized by JCat tool (http://www.jcat.de/)^46^ and then inserted to the pET‐28a(+) plasmid between the restriction enzymes sites EcoRI and BamHI. In addition, the solubility of the overexpressed vaccine protein was assessed by Protein‐Sol (https://protein‐sol.manchester.ac.uk/).^47^

### 2.8 Immune response simulation

Finally, to assess the immunological response mounted against the designed chimeric vaccine, C‐ImmSim web portal (https://kraken.iac.rm.cnr.it/C‐IMMSIM/)^48^ was deployed which mimics the natural immune environment yielded more potent immune response data of B‐cells, HTL, CTL immunoglobulins and cytokines, among others. Simulation volume and simulation steps were set at 10 and 100, respectively. Other parameters were set as default.

### 2.9 Disulfide engineering

The vaccine ensemble was also subjected to disulfide engineering to strengthen the stability and improve the folding. This was accomplished via the Disulfide by Design 2 web portal (http://cptweb.cpt.wayne.edu/DbD2/).^49^ Only the results with bond energy >5 kcal/mol were considered.

## 3 RESULTS

### 3.1 CTL epitopes

CTL epitopes obtained from MPXV as well as Omicron proteins are listed in Table 1. Only 2 CTL epitopes were obtained from Omicron proteins that were non‐toxin and non‐allergen whereas only 4 of MPXV surface glycoprotein were involved since there were many epitopes. The Comb score of predicted epitopes as well as the corresponding antigenicity were significantly high enough to be lead candidates.

**TABLE 1:** CTL epitopes extracted from both viruses along with their antigenicity, toxicity and allergenicity.

Epitope	Comb score	Antigenicity	Toxicity	Allergenicity
MPXV				
KICDINGTY	3.178	1.3504 (Probable Antigen)	Non‐Toxin	Probable Non‐Allergen
HTTCSILLR	1.383	1.2321 (Probable Antigen)	Non‐Toxin	Probable Non‐Allergen
CSSRTRKIY	2.974	0.8077 (Probable Antigen)	Non‐Toxin	Probable Non‐Allergen
CKSYIHIEY	2.7840	0.9678 (Probable Antigen)	Non‐Toxin	Probable Non‐Allergen
Omicron				
ATSRTLSYY	2.6146	0.6108 (Probable Antigen)	Non‐Toxin	Probable Non‐Allergen
LVGLMWLSY	1.3974	1.0633 (Probable Antigen)	Non‐Toxin	Probable Non‐Allergen

### 3.2 HTL epitope

The best five epitopes generated from the precursor proteins are summarized in Table 2. The percentile rank of all epitopes was <2, indicating the good binding nature. Three out of five HTL epitopes of MPXV were probable IFN‐ɣ‐secretion inducers. On the contrary, the top‐five HTL epitopes of Omicron were IFN‐ɣ inducers.

**TABLE 2:** HTL epitopes extracted from both viruses along with their antigenicity, toxicity, allergenicity and IFN‐ɣ secretion activity.

Epitope	Percentile rank	Antigenicity	Toxicity	Allergenicity	IFN‐ɣ inducer
MPXV					
NYTIDYSTVITTEEL	0.71	0.6655 (Probable antigen)	Non‐toxin	Probable non‐allergen	No
AMALYFLDVIDSEIL	0.85	0.5343 (Probable antigen)	Non‐toxin	Probable non‐allergen	Yes
MALYFLDVIDSEILY	0.86	0.5646 (Probable antigen)	Non‐toxin	Probable non‐allergen	Yes
IYIAYRNDTSFKQNT	1.60	0.7516 (Probable antigen)	Non‐toxin	Probable non‐allergen	No
CYNVSVSDASFRITL	1.80	1.2600 (Probable antigen)	Non‐toxin	Probable non‐allergen	Yes
Omicron					
FLLVTLAILTALRLC	0.38	0.6311 (Probable antigen)	Non‐toxin	Probable non‐allergen	Yes
VTLAILTALRLCAYC	0.38	0.8599 (Probable antigen)	Non‐toxin	Probable non‐allergen	Yes
RTLSYYKLGASQRVA	0.67	0.5644 (Probable antigen)	Non‐toxin	Probable non‐allergen	Yes
TLSYYKLGASQRVAG	0.67	0.4376 (Probable antigen)	Non‐toxin	Probable non‐allergen	Yes
SRTLSYYKLGASQRV	1.3	0.5526 (Probable antigen)	Non‐toxin	Probable non‐allergen	Yes

### 3.3 Linear B‐cell epitope

Surprisingly, only 3 B‐cell epitopes from 3 precursor Omicron proteins fulfilled the inclusion criteria, with each protein contributing only 1 epitope. In contrast, three epitopes were obtained from gp182 (Table 3). This is traced to the fact that MPXV surface glycoprotein is larger than the three Omicron proteins altogether.

**TABLE 3:** B‐cell epitopes extracted from both viruses along with their antigenicity, toxicity and allergenicity.

Epitope	Antigenicity	Toxicity	Allergenicity
MPXV			
KTALYHDIQLEHVEDNKDSVASLPY	0.7431 (Probable antigen)	Non‐toxin	Probable non‐allergen
GVKDDENNTVY	0.5133 (Probable antigen)	Non‐toxin	Probable non‐allergen
KIMPRVPITATEAD	0.7704 (Probable antigen)	Non‐toxin	Probable non‐allergen
Omicron			
YVYSRVKNLNSSRVP	0.4492 (Probable antigen)	Non‐toxin	Probable non‐allergen
QSCTQHQPYVVDDPCPIHFYSKW	0.4371 (Probable antigen)	Non‐toxin	Probable non‐allergen
DEAGSKSPIQYIDIGN	0.9110 (Probable antigen)	Non‐toxin	Probable non‐allergen

### 3.4 Conformational B cell epitopes prediction

**FIGURE 2:**
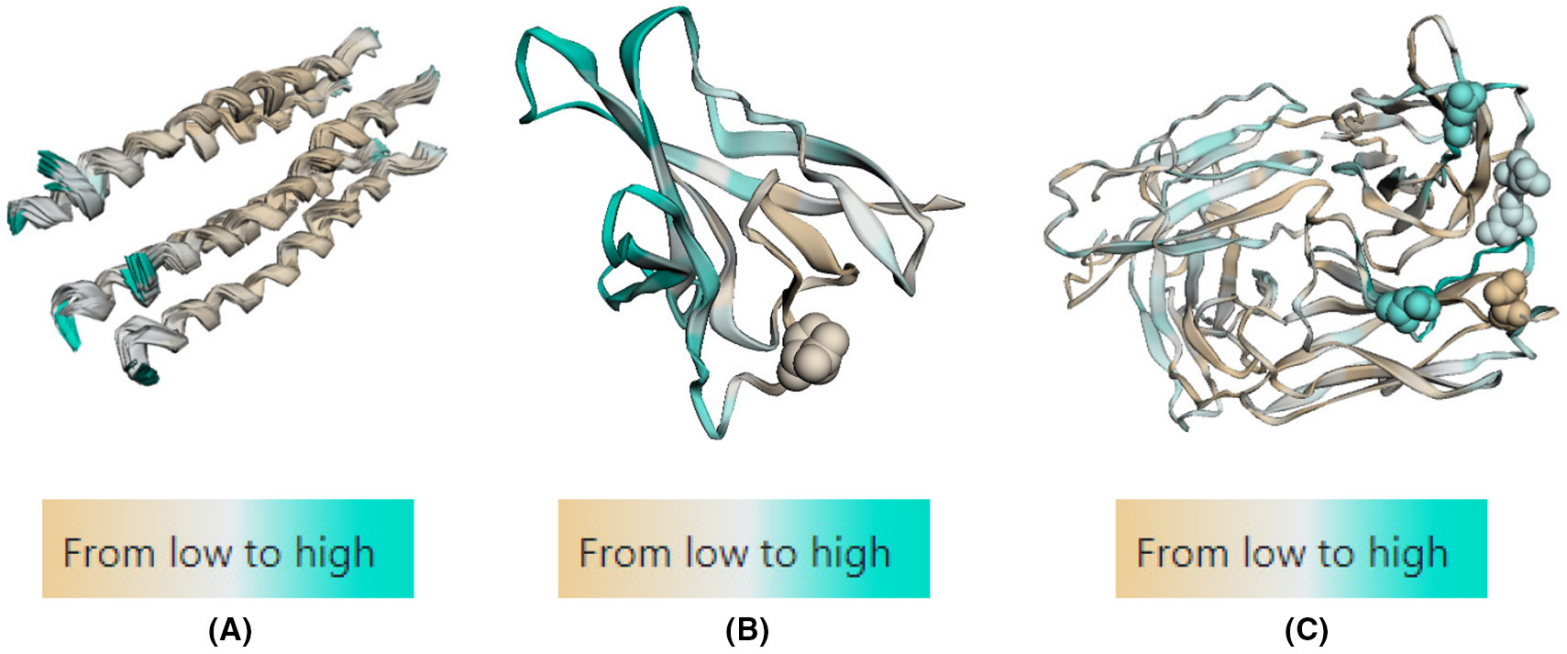
Conformational B cell epitopes of E (A) protein, ORF8 (B) protein, and M (C) protein. potnetial conformational eptiopes are shown in cyan, while.

The predicted conformational B cell epitopes from all selected antigens are illustrated in Figure 2. ORF8 was the antigen with greatest number of conformational B cell epitopes followed by M protein as indicated by the cyan colour. E antigen, however, exhibited small portion at the N‐terminal which could be traced to its helical configuration. Besides, glycosylation sites were found in ORF8 and M antigen as depicted as spheres.

### 3.5 Vaccine construction

**FIGURE 3:**
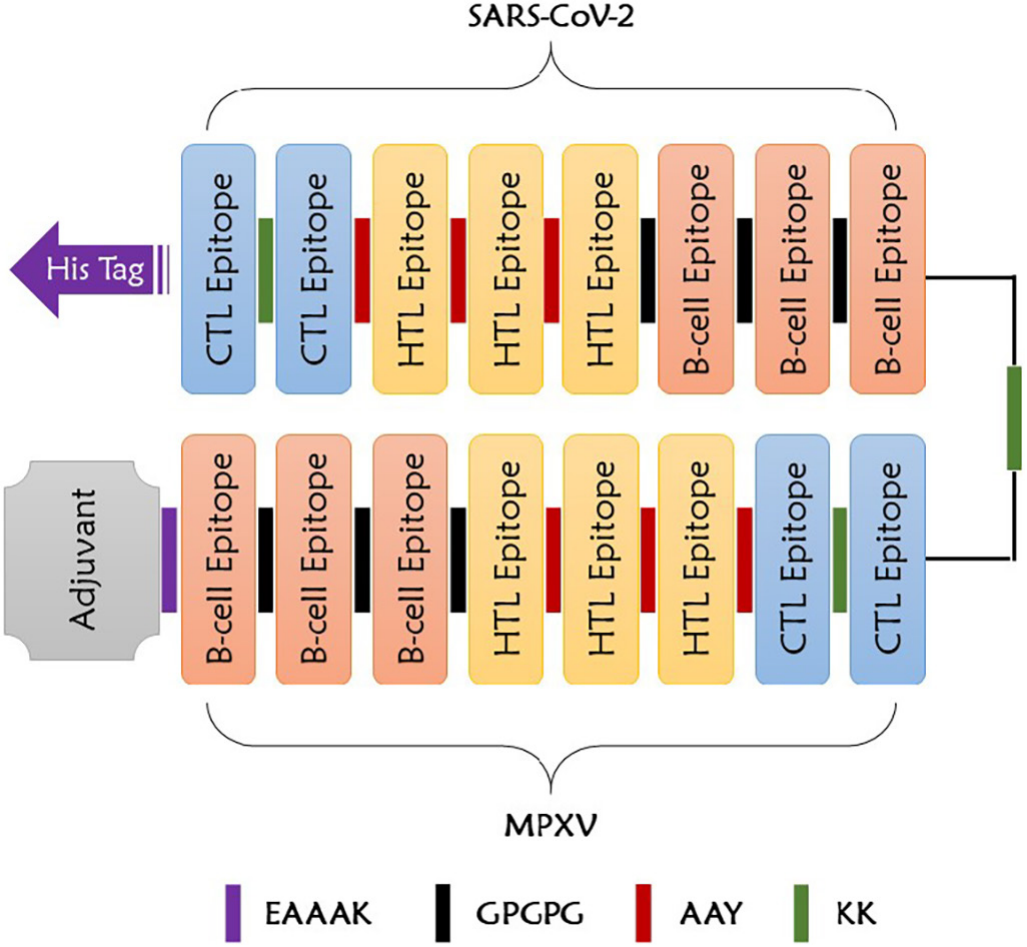
Multi‐epitope vaccine linking strategy.

In order to form multi‐epitope vaccine construct, 3 B‐cell epitopes, 3 HTL epitopes and 2 CTL epitopes from the two viruses were joined together to the adjuvant β‐Defensin (45 amino acids; GIINTLQKYYCRVRGGRCAVLSCLPKEEQIGKCSTRGRKCCRRKK) in the N‐terminal and to 6 His residues (His tag) in the C‐terminal via different linkers as elucidated in Figure 3. These linkers enhance the immune response formed and increase the recruitment of various immune cells to the vaccination site. Notably, the antigenicity of the vaccine construct was 0.5435 with no probable allergenicity.

### 3.6 Secondary structure prediction

**FIGURE 4:**
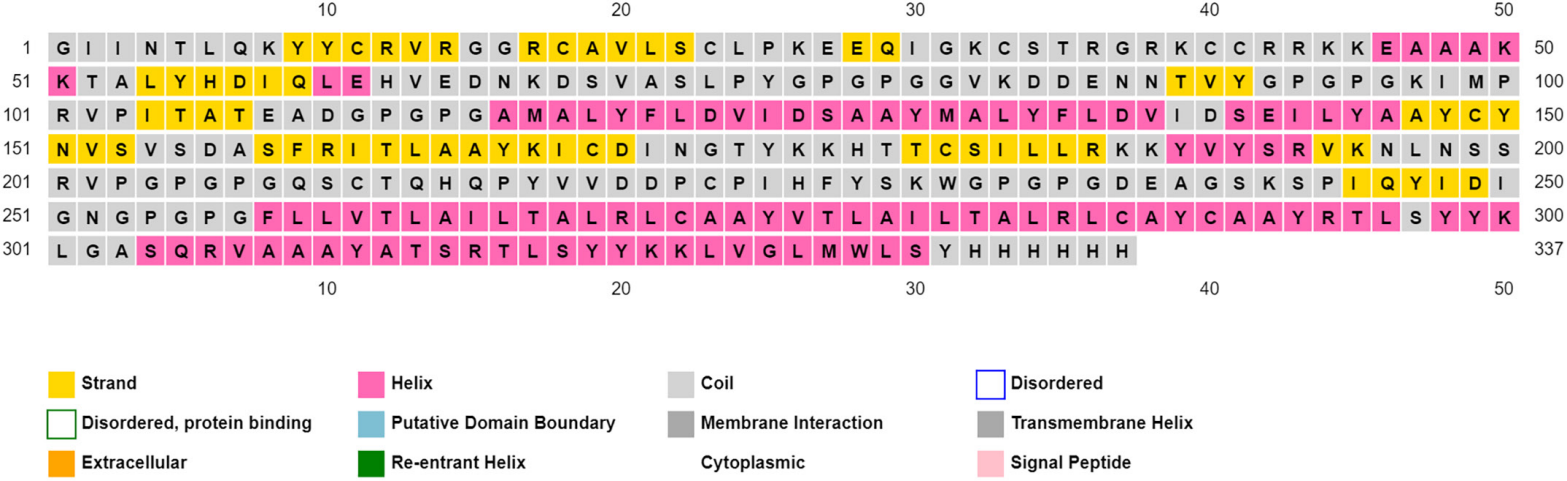
Predicted secondary structure of the chimeric vaccine construct.

Protparam descriptions unveiled a molecular weight of 36,955 Da, isoelectric point of 9.09 which means an overall alkaline property as mirrored by the higher positively‐charged residues in comparison with negatively‐charged ones (39 vs. 25). The vaccine construct was predicted to pose >10 h half‐life in vitro (*E. coli*) and about 30 h in vivo (mammalian reticulocytes). In addition, with an instability index of 35.12 and an aliphatic index of 84.87, the construct is classified as stable and good thermos ability profile respectively. Grand average of hydropathicity (GRAVY) was calculated to be −0.159 indicating the slightly hydrophilicity of the overall protein. Collectively, this valorizes the quality of constructed vaccine. PSIPRED described the amount of each secondary structure: 33% α‐helix, 18% β‐pleated sheets while the coils content represented ~49% as depicted in Figure 4.

### 3.7 Tertiary Structure prediction, refinement and validation

**FIGURE 5:**
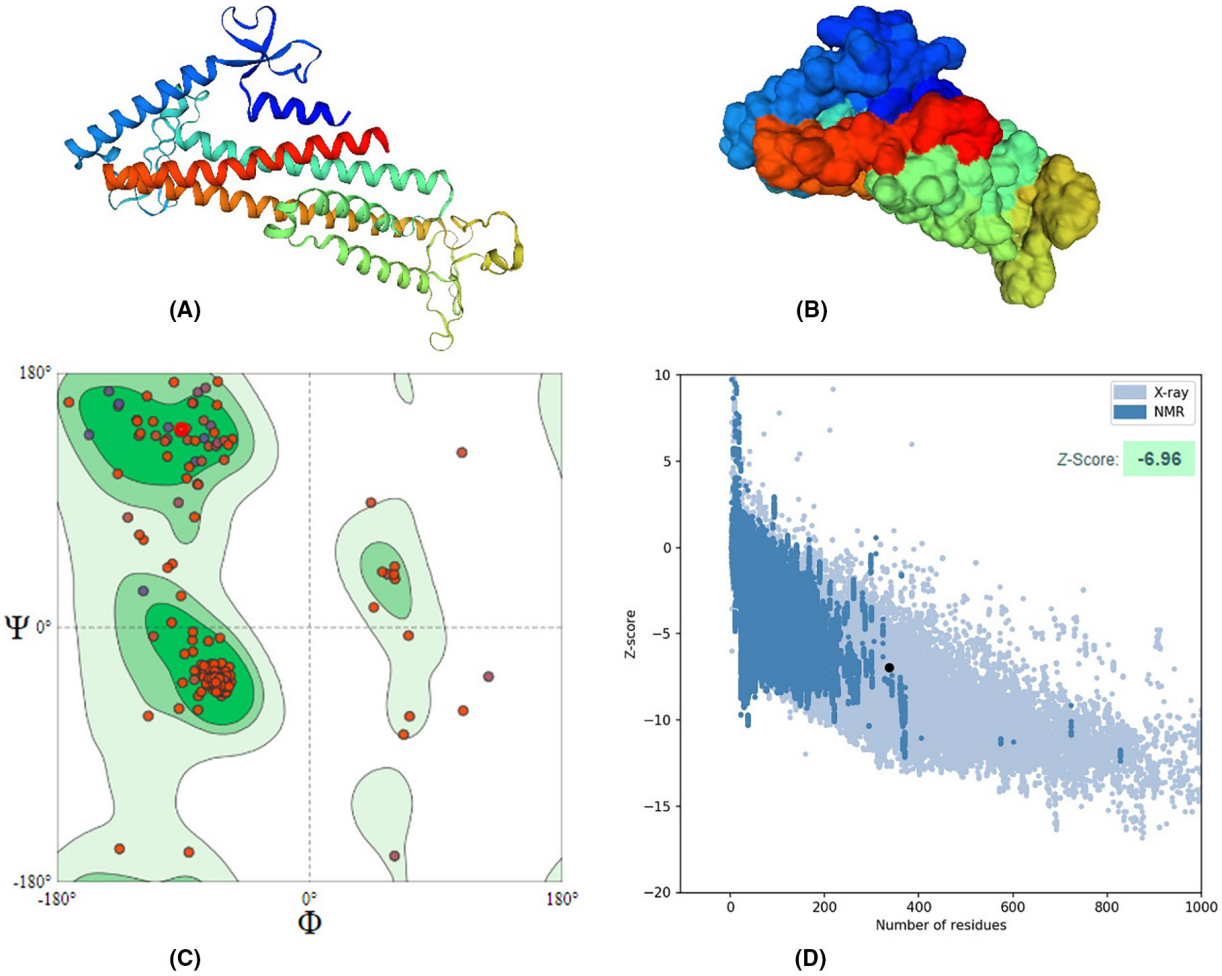
3D structure of the chimeric vaccine shown in cartoon (A) and surface (B) along with its validation by Ramachandran plot (C) and ProsA (D) Z‐score of the constructed vaccine falls within x‐ray crystallographic structures, a very accurate and high‐resolution technique for protein structure elucidation, with a high value (−9.96) signifying its high quality.

The vaccine protein was modelled using RoseTTafold platform and then validated by Ramachandran plot and other assessment parameters generated from SWISSMODEL in addition to ProsA server. The modelled architecture was refined through the GalaxyRefine webserver. The refined structure had a MolProbity score of 1.76, significantly lower than the model's 3.21. Furthermore, the clash score was also reduced (from 166.48 to 6.54), whereas Ramachandran's favourite regions were elevated (94.04% vs. 90.75%). Also, the Z score of the ProsA validation server was −6.96 and within the X‐ray crystallography zone, reflecting the powerful degree of refinement by the GalaxyRefine webserver Figure 5.

### 3.8 Molecular docking

**FIGURE 6:**
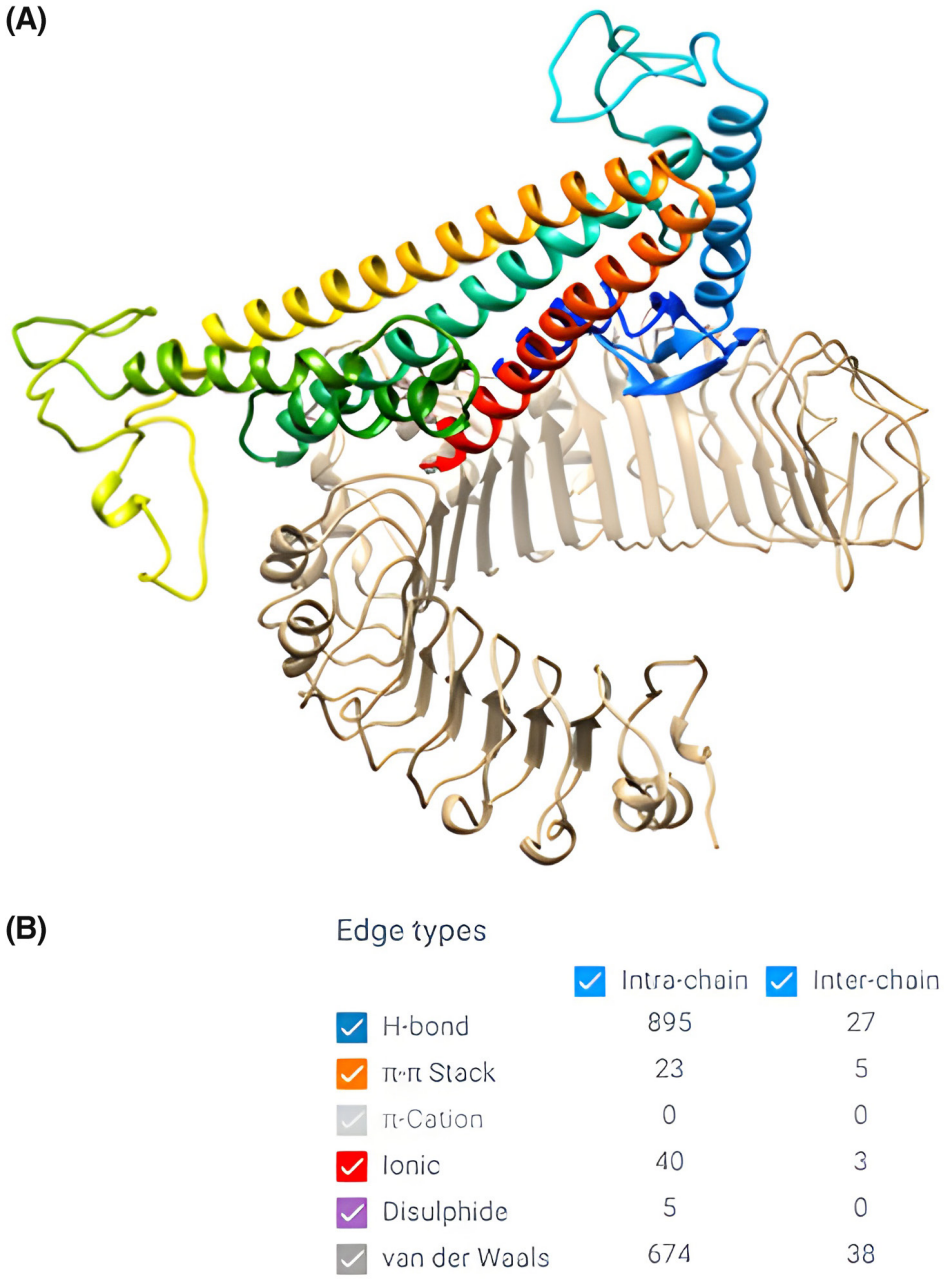
Molecular docking of the chimeric vaccine to TLR‐2 such that TLR‐2 is shown in light gold whereas the vaccine in rainbow (A) and the detailed protein–protein interaction pattern (B).

The lowest free energy of binding between the two proteins was −1169.8 kcal/mol. Such strong binding energy is accounted for by the detailed protein–protein interactions predicted using the RING tool. The protein–protein interaction generated 27 H‐bonds, 5 π‐ π stack, 3 ionic bonds, and 38 van der Waals interactions. This indicates the highly immunogenic potency of the constructed vaccine, as demonstrated by its strong binding to the innate immune cell receptor TLR‐2 (Figure 6).

### 3.9 MD simulation

The stability and flexibility of a vaccine are critical factors in its efficacy and ability to provide long‐lasting protection against a virus. The MD simulations conducted for the constructed vaccine have demonstrated that it is a highly stable and flexible vaccine. The RMSD diagram shows that the vaccine is stable from the first 20 ns, indicating that the vaccine maintains its folded structure throughout the simulation. This stability is crucial for the vaccine to be effective in triggering an immune response and preventing the virus from infecting cells.

Moreover, the RMSF analysis revealed that the vaccine construct has minimal residual fluctuations, indicating that it is a well‐structured and stable vaccine. However, some fluctuations were observed between positions 270 and 300, which are not unexpected as the vaccine is undergoing minor structural changes during binding the TLR. Additionally, the high flexibility loops observed at the N‐terminal and C‐terminal of the vaccine construct may aid in the recognition and binding of the viral antigen.

The Rg of the vaccine ensemble displayed a highly stable pattern. In other words, Rg values were shown to be around ~3 nm during the whole MD simulation course (100 ns). This indicates the reduced conformational changes and compact configuration of the vaccine ensemble.

**FIGURE 7:**
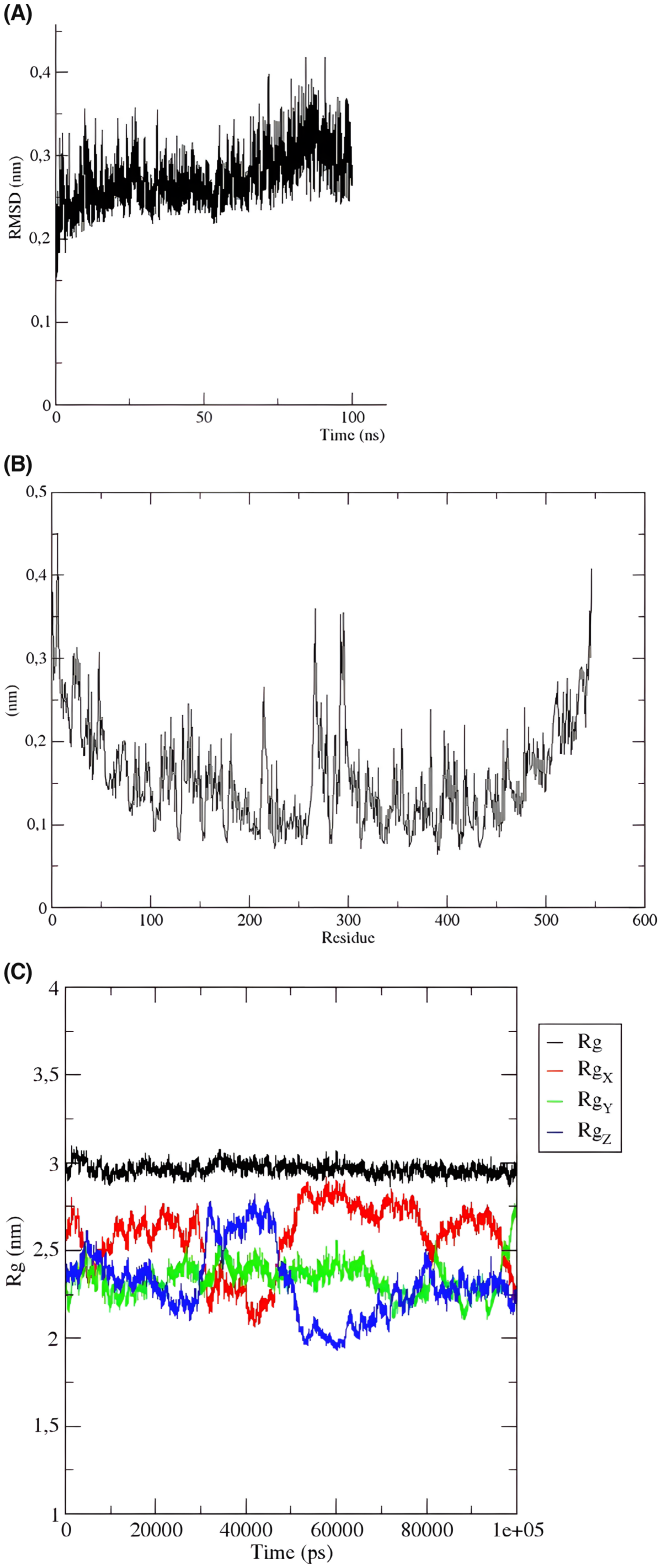
MD simulations findings of the vaccine construct for 100 ns. RMSD (A), RMSF (B) were elucidated.

Overall, the results of the MD simulations provide strong evidence that the constructed vaccine is a highly stable and flexible vaccine, which is essential for its efficacy and ability to provide long‐lasting protection against the virus (Figure 7A,B).

Concerning free energy of binding was calculated for the vaccine‐TLR2 complex and summarized in Table 4. The results showed that the total free binding energy was calculated to be −55.63 kcal/mol. Such high energy signifies the strong binding between the constructed vaccine and TLR2. Most of this energy was contributed by electrostatic attraction with −981 kcal/mol.

**TABLE 4:** MM/GBSA calculation of the vaccine‐TLR2 complex.

Parameter	Energy (kcal/mol)
VDW	−117.37
ELE	−981.21
GB	1057.49
SA	−14.55
Total	−55.63

Abbreviations: ELE, electrostatic potentials; GB, polar solvation free energies predicted by the generalized born model; SA, Nonpolar contribution to the solvation free energy calculated by an empirical model; VDW, Van der Waals potentials.

### 3.10 Vector preparation and virtual cloning

**FIGURE 8:**
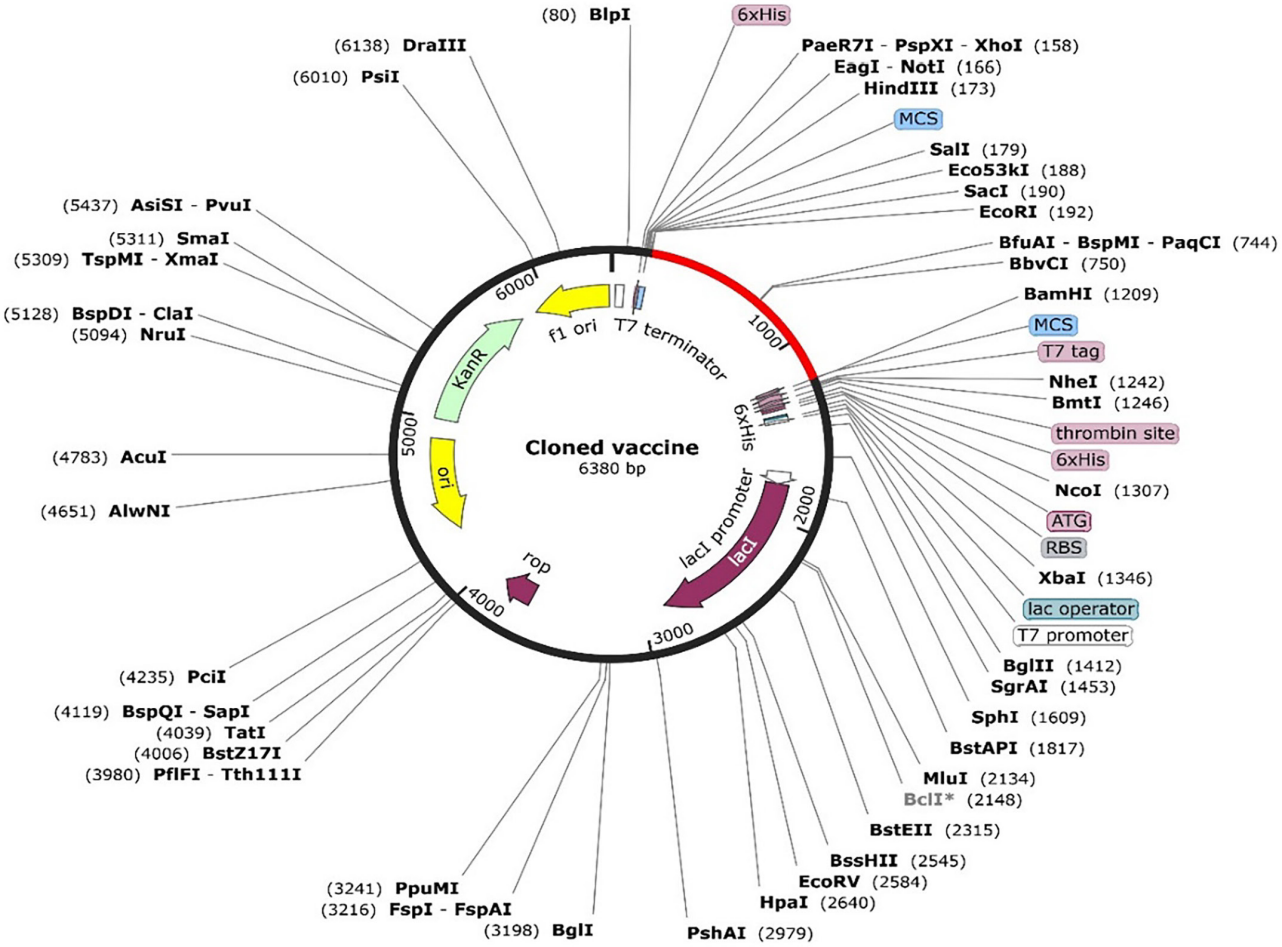
Cloned vaccine in the pET‐28a plasmid. The vaccine sequence is shown in red while the rest of plasmid in black.

Codon content was diminished upon codon optimization using the JCat tool from 57 to 50%. The CAI value of the optimized sequence was 0.39, indicating an acceptable expression probability in the E. coli K12 expression system. The optimized sequence (1011 bases) was inserted in the region between the restriction enzymes EcoRI and BamHI of the plasmid, as illustrated in Figure 8. This provides a ready‐to‐use plasmid, pET‐28a (+), containing the vaccine construct to be applied in a wet‐lab setting.

### 3.11 Vaccine construct solubility

**FIGURE 9:**
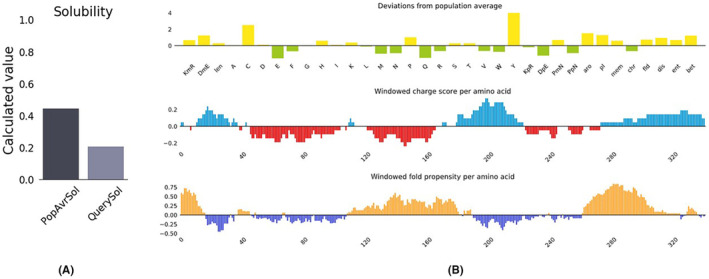
Protein solubility profile of the recombinant vaccine (A) in addition to its deviation, charge and fold propensity (B).

The protein‐sol was used to calculate the solubility of our vaccine construct (https://protein‐sol.manchester.ac.uk/results/solubility/run‐a530cad3673133be9ea5/results.html).Solubility of the inclusion body proteins is an extraordinary step in vaccine biotechnology since soluble proteins facilitate their downstream isolation and purification. The vaccine protein construct is found to be soluble (calculated score 0.4) when overexpressed in E. coli, as shown in Figure 9. This ensures the ease of downstream isolation and purification steps for the overexpressed vector.

### 3.12 C‐immune simulation

**FIGURE 10:**
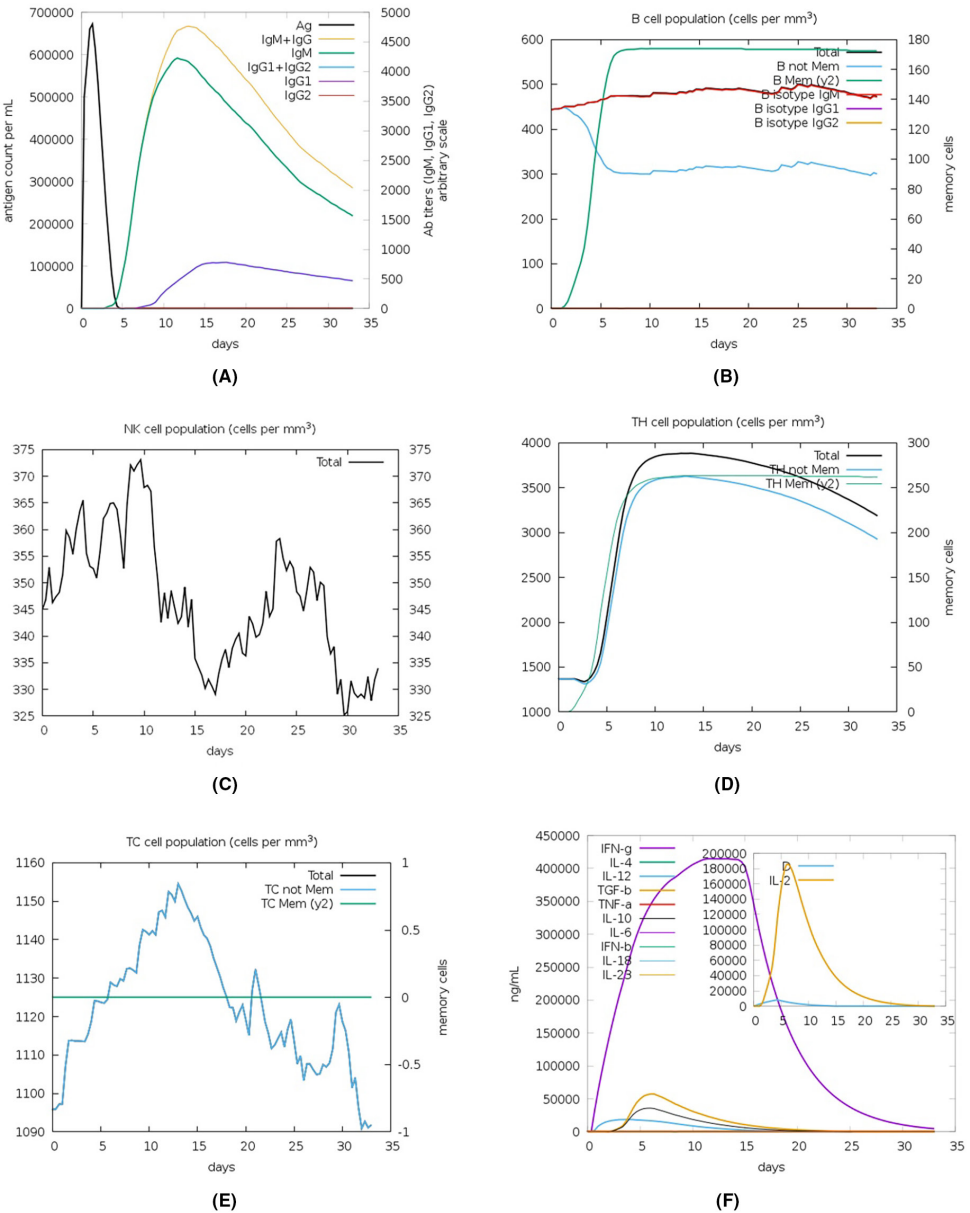
Immune response simulation profile of the chimeric vaccine. The response involves antigen levels (A), circulating antibodies levels (B), NK cells (C), HTL (D), CTL (E) and the predominant cytokine (F).

Computational immune simulation (C‐ImmSim) server was deployed for predicting the potential immune response against the constructed chimeric vaccine. The designed vaccine exhibited satisfactory immunosimulation upon injection, as indicated by the in silico immune simulation profile (Figure 10). Indeed, levels of antibodies of the two types, IgM and IgG, were rapidly elevated after vaccine injection. The total B cells were also increased with IgM‐secreting and IgG‐secreting B‐cells were consistently plateaued and gradually declined after vaccine injection. In contrast, memory B‐cells rose after a while, but the increase was steep, providing probable long‐term memory. Natural killer (NK) cells exhibited a drop in the 3rd week (a drop between 2 peaks in the first 2 weeks and the last week of the month after injection). Of the total elevated HTL cells, memory HTL was predominant. The dominant type of increased CTL was the cytotoxic, whilst the dominant cytokine secreted was IFN‐ɣ mirroring the antiviral direction the immune system plays toward the vaccine construct.

### 3.13 Disulfide engineering

**FIGURE 11:**
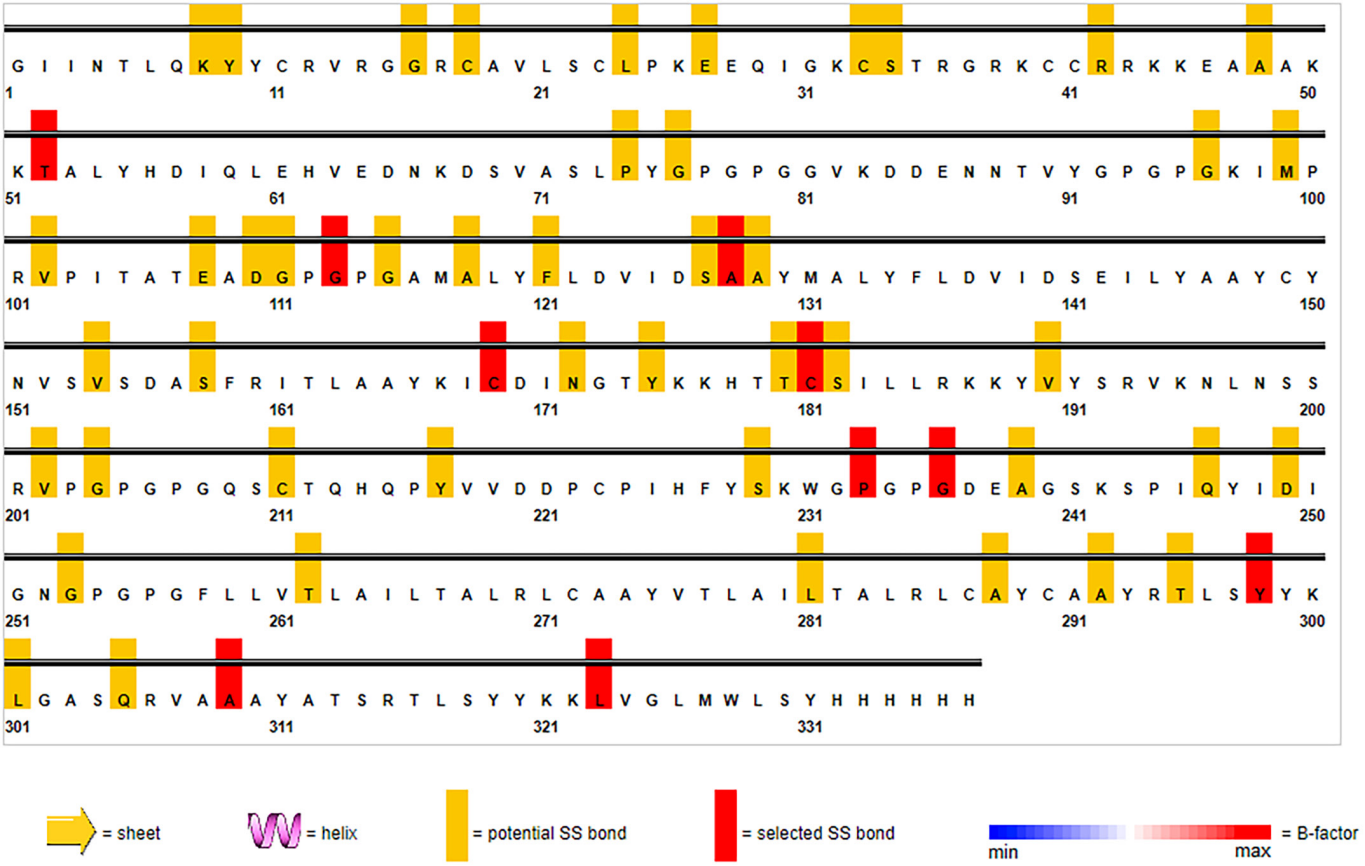
2D visulaization of disulfide bond forming candidates of the vaccine ensemble.

**FIGURE 12:**
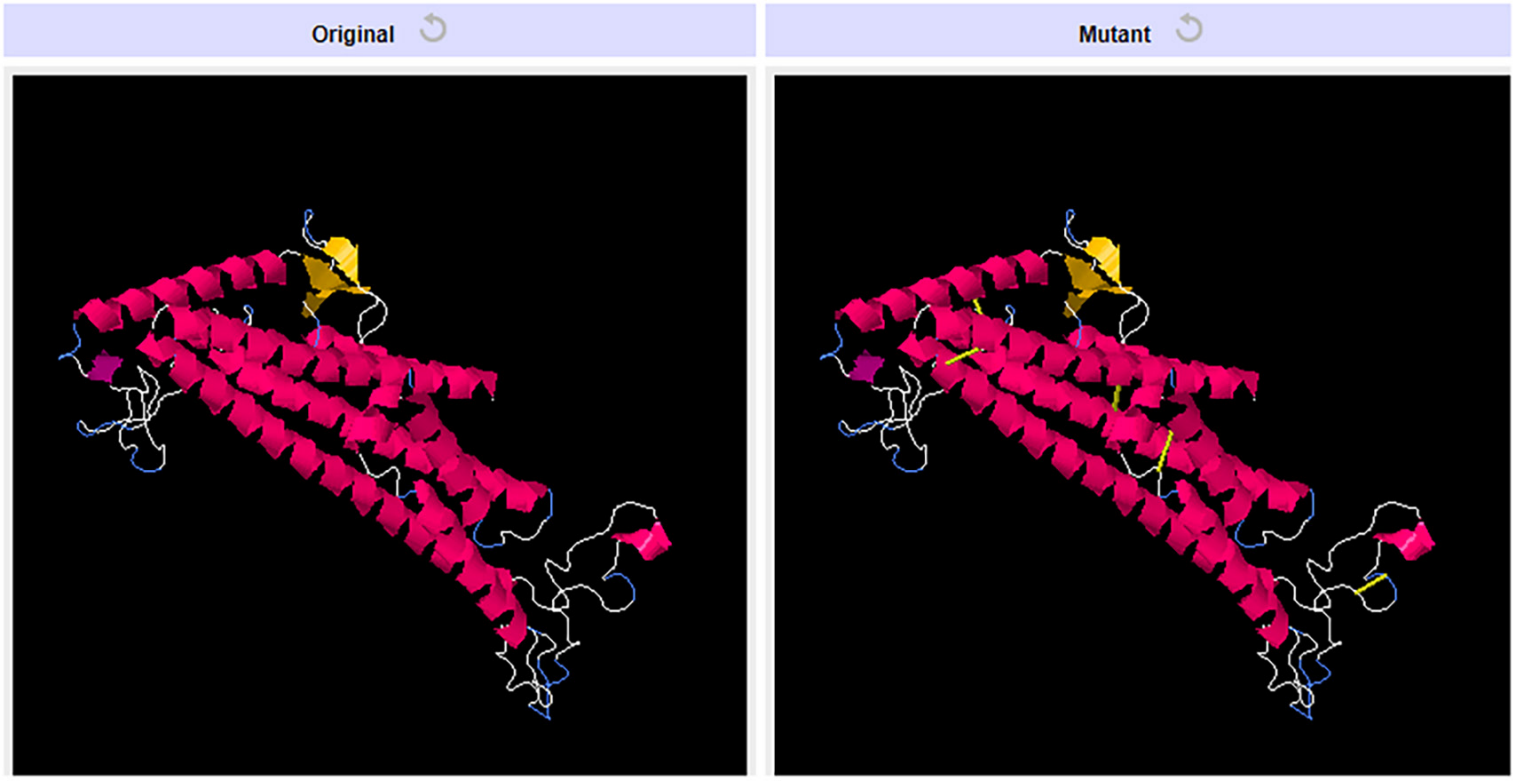
3D Visulaization of the mutant vaccine ensemble with the five selected amino acid pair.

The vaccine ensemble upon subjected to disulfide engineering resulted in numerous possible disulfide engineering. Only the top five mutants with energy value greater than 5 kcal/mol were selected and listed in Table 5. The position of the amino acid pairs selected for disulfide engineering is depicted in Figure 11 in the 2D visualization. Also, the 3D structure of the mutant version containing all the five engineered bonds is illustrated in Figure 12.

**TABLE 5:** Top 5 disulfide bond forming amino acid pairs along with the corresponding angle and bond force.

First AA	Second AA	χ3	kcal/mol	B‐Factor
Thr52	Gly113	−113.73	6.49	0
Pro233	Gly236	−72.68	6.14	0
Ala128	Leu323	110.83	6.04	0
Tyr298	Ala309	−87.95	5.83	0
Cys169	Cys181	108.37	5.34	0

## 4 DISCUSSION

Currently, COVID‐19 has become a global pandemic affecting nearly every country in the world. Recent statistical data indicates that over 651 million confirmed cases with a fatality ratio of approximately 1% have been reported. Despite the availability of antiviral drugs and neutralizing antibodies, the waves of infection continue to persist. Furthermore, the administration of mRNA vaccine boosters has not achieved the intended efficacy, and some countries have begun to implement herd immunity as a complementary strategy to address this ongoing outbreak.^50^, ^51^ MPXV on the other hand, albeit it has spread from Africa to Europe and North America, is less virulent than COVID‐19 but the light has not been highlighted to it as same as COVID‐19. Given that no available vaccine to such pandemic is aggravating the situation since the medical society has not yet been recuperated.^52^ Immunoinformatics has emerged as a valuable and powerful tool in the field of immunology, allowing researchers to effectively assess the antigenicity of specific B‐cell and T‐cell epitopes. In addition, the ability to perform complete in silico design, efficacy testing, and cloning enables significant cost and time savings, which are particularly critical during pandemics such as the current outbreaks of COVID‐19 and MPXV.^53^ To the best of our knowledge, the immunoinformatics approaches employed to date deal with only one pathogen at a time, but no chimeric vaccine has been constructed to serve against SARS‐CoV‐2 as well as MPXV. Hence, this was the goal of the present study.

Three B‐cell epitopes, three HTL epitopes, and two CTL epitopes were selected to be used further for vaccine construction. HTL epitopes selected proved their activity as IFN‐ɣ inducers and all of the selected epitopes were probable antigen as described by VaxiJen 2.0 server, non‐toxin and non‐allergen confirming the safety of the chosen epitopes. After joining using specific peptide linkers, the secondary and tertiary structures of the proteins were predicted and validated. Then, docking TLR‐2 revealed the high probability of mounting an immune response upon in vitro testing since the docking score was −1169.8 kcal/mol. TLRs, which are commonly associated with identifying pathogen membrane constituents, have also been found to facilitate innate immune responses to specific viral pathogens, utilizing their presence on the cell surface, particularly TLR2.^54^ Afterwards, the vaccine sequence was cloned to the highly overexpressed plasmid pET‐281(+), and its solubility upon isolation and purification steps was acceptable. pET‐28a is the most popular expression vector in the biotech market (mentioned in >40,000 published articles). It has a copy number of 40, indicating the high yield as well as ease of downstream purification of the recombinant protein.^55^ Lastly, the immune simulation profile gave excellent findings involving high B‐cells, HTL, CTL, NK cells and macrophages increase and response in addition to the predominant IFN‐ɣ secretion which poses antiviral activity. It has been documented that IFN‐ɣ and NK cells are the first‐line defences against the invading viruses including SARS‐CoV‐2, reflecting the important role in virus eradication.^56^ All these findings emphasize the potency, efficacy, safety, and, thus, the eligibility of the chimeric vaccine construct as an untested strategy to limit the simultaneous pandemics. Many previous works reported the application of immunoinformatics tools to design and test the potency of the vaccine against many targets of the two viruses. Baloch et al.^34^ designed a vaccine construct against SARS‐CoV, SARS‐CoV‐2 and MERS by targeting Spike glycoprotein as the epitopes precursor. Sharma et al.^57^ constructed a COVID‐19 vaccine by targeting Omicron receptor‐binding domain of Spike protein. Similarly, immune epitopes extracted from sequence of E, M, N and Spike proteins were utilized for another COVID‐19 vaccine design.^58^ Even RNA‐dependent RNA polymerase‐targeting COVID‐19 vaccine was contrived in similar fashion.^59^

Likewise, various studies suggested the employment of immunoinformatics to contrive a vaccine specific for MPXV against different protein precursors such as cell surface‐binding protein,^60^ L1R, B5R, and A33R,^61^ COP‐A44L and COP‐B7R^53^ and another antigenic proteins,^62^ among others. We used ORF8 besides E and M proteins to extract the epitopes in SARS‐CoV‐2 whereas gp182 was used as the precursor protein in MPXV and the designed vaccine demonstrated its potency (antigenicity 0.543) and safety (non‐allergen) which deserves experimental validation.

Clinical translation of chimeric vaccines is scarce. For example, Cervarix, a licensed virus‐like particle vaccine against diseases associated with HPV16 and HPV18, is a multivalent vaccine targeting multiple strains of the same pathogen (human papillomavirus, HPV).^63^ Similarly, Q‐VAX is a licensed formalin‐inactivated whole‐cell vaccine against *Coxiella burnetii*, the causative agent of Q fever.^64^ These studies proved the explitation of chimeric vaccine but for different strains of the same pathogens. The present study designed chimeric vaccine against two distinctly viral pathogens, namely SARS‐CoV‐2 and MPXV.

## 5 CONCLUSION

The development of effective vaccines against emerging viruses is crucial in the fight against infectious diseases. In this study, a chimeric vaccine was designed to provide protection against two of the most concerning emerging viruses, SARS‐CoV‐2 and MPXV. The chimeric vaccine construct was designed to contain multiple immunogenic epitopes capable of stimulating B‐cells, HTL, CTL, and IFN‐ɣ, which are essential components of the immune response against viral infections. The vaccine was designed to be highly potent and safe, with the potential to induce a strong immune response against both viruses simultaneously. To facilitate downstream purification protocols, the vaccine was inserted into the pET‐28a vector, which is predicted to produce a soluble protein product. The results of this study are promising and suggest that the chimeric vaccine construct has the potential to be an effective tool in the fight against SARS‐CoV‐2 and MPXV. Also, the methodology employed in the current study is innovative to design multivalent vaccine against more than one pathogen. Further testing and consideration are necessary to fully evaluate the efficacy and safety of this novel vaccine. In the experimental setting, the immunogenicity of the injected vaccine can be assessed via different tools, i.e. FACS (Fluorescence‐Activated Cell Sorting) to decipher the changes in immune cells pattern (for cell‐mediated immunity) and End‐point ELISA (Enzyme‐linked immunosorbent assay) which involves measuring the binding of antibodies to specific antigens reflecting the level of immune response generated by the vaccine. However, the potential benefits of the chimeric vaccine construct warrant further investigation and development, as it has the potential to provide much‐needed protection against two of the most concerning emerging viruses.

## AUTHOR CONTRIBUTIONS

**Haitham Al‐Madhagi:** Conceptualization (equal); data curation (equal); methodology (equal); visualization (equal); writing – original draft (equal). **Adeela Kanawati:** Data curation (lead); investigation (equal). **Zaher Tahan:** Supervision (lead); writing – review and editing (lead).

## FUNDING INFORMATION

No funding was used in this study.

## CONFLICT OF INTEREST STATEMENT

The authors confirm that there are no conflicts of interest.

## Data Availability

All data generated or analysed during this study are included in the article.
